# Surgical Resection of Superior Vena Cava Saccular Aneurysm

**DOI:** 10.1016/j.atssr.2024.04.005

**Published:** 2024-04-24

**Authors:** María Alejandra Rodríguez Brilla, Kevin Daniel Kausen, Ariana Ollé, Victor Bautista-Hernandez

**Affiliations:** 1 Division of Cardio-thoracic Surgery, Michael E. DeBakey Department of Surgery, Baylor College of Medicine at Christus Children’s Hospital of San Antonio, Texas; 2 Faculty of Medicine and Health Sciences, University of the Incarnate Word School of Osteopathic Medicine, San Antonio, Texas; 3 Faculty of Medicine and Health Sciences, University of Barcelona, Barcelona, Spain

## Abstract

Aneurysms of the superior vena cava are rare vascular malformations of systemic veins. This report presents the case of a 27-year-old woman with an incidental finding of mediastinal shadow widening on the chest roentgenogram that was confirmed by computed tomographic angiography to be a superior vena cava saccular aneurysm >4 cm in diameter. Surgical resection was recommended on the basis of aneurysmal size and shape and was performed through median sternotomy by using cardiopulmonary bypass. The postoperative period was uneventful. During follow-up visits she remained asymptomatic. Saccular aneurysm surgery was recommended to prevent associated complications, including rupture, thrombosis, or venous obstruction.

Superior vena cava (SVC) aneurysms are among the rarest causes of mediastinal masses of venous origin. Most of the cases are asymptomatic and discovered incidentally as mediastinal shadow widening on a chest roentgenogram.^1^ SVC aneurysms can be classified morphologically as fusiform or saccular.^1^ In the literature, a total of 72 reports have documented SVC aneurysms, with only 25 of these reports describing the saccular variety.^2^

Recommended treatment of fusiform aneurysms is conservative management with anticoagulation therapy and orderly observation.^3^ Saccular aneurysms are associated with an increased risk of thrombus formation, venous obstruction, and potential rupture.^3^ Therefore, surgical resection is the recommended treatment considering the aneurysmal size and morphologic features and the reduction of life-threatening complication risk.^3^

A 27-year-old female patient presented with a 5-day history of right upper quadrant pain and nausea without vomiting. She had no reported past medical history. The patient received a diagnosis of cholecystitis. A chest roentgenogram was obtained and revealed mediastinal shadow widening (Figure 1). This finding prompted chest and abdominal computed tomographic (CT) angiography, which showed an SVC saccular aneurysm measuring 4.2 × 4.5 cm with contribution from the innominate vein and an enlarged or aneurysmatic azygos vein (Figure 2), without evidence of other venous aneurysms elsewhere.

**Figure 1:**
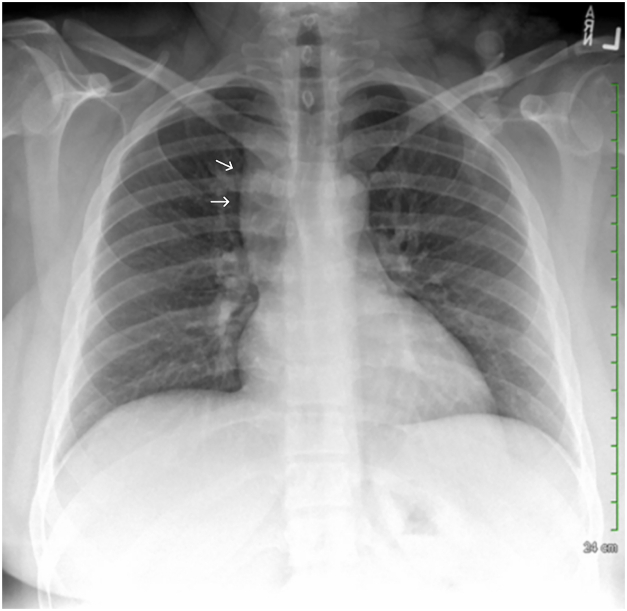
Chest roentgenogram. Posteroanterior view showing a right-sided mediastinal shadow widening at the level of the ascending aorta (arrows).

**Figure 2:**
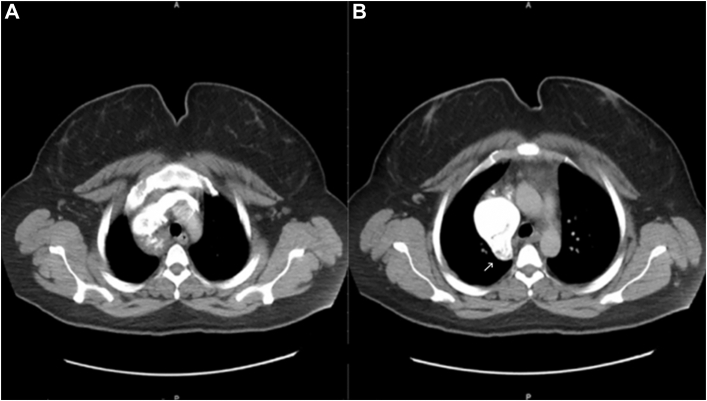
Computed tomographic angiography. Axial view showing (A) the innominate vein and (B) the azygos vein contribution to the superior vena cava saccular aneurysm (arrow).

The decision was made to perform surgical resection considering the aneurysmal size and morphologic features. The chest cavity was opened through a median sternotomy. Cardiopulmonary bypass (CPB) was used, cannulating the right atrium appendage, the innominate vein, and the inferior vena cava. The clamps were placed on the distal and proximal SVC after innominate vein and right atrium appendage cannulation for aneurysmal dissection. SVC reconstruction was performed with a pericardial patch, and the dilated azygos vein entering the aneurysm was occluded with pledgeted sutures.

The patient’s recovery was uneventful, and she was discharged on day 3. Postoperative imaging revealed significantly reduced mediastinal shadow widening on the chest roentgenogram (Figure 3A). Repeat CT angiography showed an occluded azygos vein with pericardial patch placement at the site of the former aneurysm (Figure 3B). The 8-month follow-up imaging demonstrated no aneurysmal recurrence. The patient continued treatment with aspirin.

**Figure 3:**
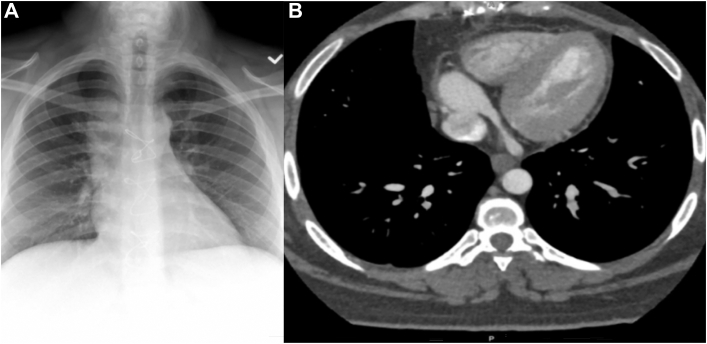
Postoperative imaging. (A) Posteroanterior chest roentgenogram showing reduced right-sided mediastinal shadow widening. (B) Axial computed tomographic angiography showing the resected azygos vein and a pericardial patch on the superior vena cava.

## Comment

SVC aneurysms are rare causes of mediastinal masses arising from the venous system. The precise mechanism by which this vascular malformation forms has not been elucidated. SVC aneurysms are usually primary, and their origin can be attributed to congenital abnormalities or degenerative processes affecting the venous wall.^4^ Secondary aneurysms result from external factors, including inflammation, infection, and mechanical trauma.^4^ There has been an association with cystic hygromas because of the similar embryologic origin of the venous and lymphatic systems.^3^^,^^5^

The morphologic features of SVC aneurysms classifies them as saccular or fusiform.^3^ Fusiform SVC aneurysms comprise the most common variety of this vascular malformation, with only a few existing reports in the literature of the saccular variety.^6^ SVC aneurysms are usually asymptomatic, or they can manifest with nonspecific symptoms such as dyspnea and chest pain, thus making them incidental findings during workup imaging for other indications in most cases.^2^ The current standard of care imaging modality for SVC aneurysm diagnosis is CT or magnetic resonance imaging; CT is useful in aneurysmal morphologic characterization and in identification of calcifications and filling defects within the aneurysm.^2^

Treatment depends on the aneurysmal characteristics and morphologic features. For fusiform SVC aneurysms, the recommended treatment is conservative management with oral anticoagulation or antiplaque therapy and periodic imaging, considering the low risk of associated complications.^2^ Saccular aneurysms that are symptomatic, larger than 40 mm, enlarging, or that contain thrombus have been advocated to require radical surgical management.^2^ Open surgical repair is performed through a median sternotomy or right thoracotomy, with subsequent aneurysmal resection, primary suturing of the neck, and reconstruction of the SVC through the placement of a bovine pericardial patch.^2^ CPB is used for managing complex saccular aneurysms, including cases with thrombus formation, calcification, and venous obstruction.^2^^,^^4^ The endovascular approach, through an endovascular balloon-protected transcatheter thrombin injection, is an alternative treatment for high-risk patients who are unable to undergo surgical resection.^2^^,^^6^ Another endovascular approach involves occlusion of the right internal thoracic vein with coils, followed by deployment of an uncovered stent in the SVC, with coil occlusion of the neck of the sac through stent meshes.^2^

In our case, surgical resection was recommended, considering the saccular aneurysm morphologic features and the size, measuring 4.2 × 4.5 cm. The median sternotomy approach was chosen because it provides direct and wide access to the mediastinum and allows cannulation during CPB. Cannulation was performed on the right atrium appendage, innominate vein, and inferior vena cava, and clamps were placed on the distal and proximal SVC after cannulation for aneurysmal dissection. The dilated azygos vein entering the aneurysm was occluded with pledgeted sutures for occlusion of this considerable aneurysmal contributor as well as for reinforcing the posterior wall of the aneurysm. SVC reconstruction was performed with a pericardial patch to decrease the risk of stenosis.

In conclusion, although SVC saccular aneurysms are rare vascular malformations of the mediastinal systemic veins, they are associated with an increased risk of life-threatening complications. Therefore, surgical resection of saccular SVC aneurysms is recommended. This report describes the case of a 27-year-old woman with an incidental finding of a saccular SVC aneurysm who underwent successful surgical repair, thus highlighting the importance of including vascular malformations such as SVC aneurysms in the differential diagnosis of incidentally found mediastinal masses.

## References

[1] Koga S., Ikeda S., Sanuki Y., Ninomiya A., Izumikawa T., Miyahara Y. (2006). A case of asymptomatic fusiform aneurysm of the superior vena cava detected by magnetic resonance imaging. Int J Cardiol.

[2] Morales M., Anacleto A., Ferreira J., Greque V., Souza A., Wolosker N. (2021). Saccular superior vena cava aneurysm: case report and comprehensive review. Ann Vasc Surg.

[3] Jacobson A., Ailiani R., Paramesh V. (2019). A rare case of superior vena cava saccular aneurysm. CASE (Phila).

[4] Varma P.K., Dharan B.S., Ramachandran P., Neelakandhan K.S. (2003). Superior vena caval aneurysm. Interact Cardiovasc Thorac Surg.

[5] Haddad R., Joni H., Geske J. (2014). Images in vascular medicine. Twenty-eight years later: a case of superior vena cava aneurysm secondary to cystic hygroma. Vasc Med.

[6] Janczak D., Skiba J., Gemel M., Mak M., Ziomek A., Malinowski M. (2016). Giant saccular superior vena cava aneurysm-a rare and difficult clinical case. J Thorac Dis.

